# Stress Suppression Design for Radiofrequency Microelectromechanical System Switch Based on a Flexible Substrate

**DOI:** 10.3390/ma17164068

**Published:** 2024-08-16

**Authors:** Kang Wang, Zhaoer Chai, Yutang Pan, Chuyuan Gao, Yaxin Xu, Jiawei Ren, Jie Wang, Fei Zhao, Ming Qin, Lei Han

**Affiliations:** 1 Key Laboratory of MEMS of the Ministry of Education, Southeast University, Nanjing 210096, China; epiphanyer@163.com (K.W.); 220221727@seu.edu.cn (Y.P.); 230238403@seu.edu.cn (C.G.); mqin@seu.edu.cn (M.Q.); 2 National Engineering Research Center of Communication Software and Asic Design, China Electronics Technology Group Corporation 54th Research Institute, Shijiazhuang 050081, China; ohyeahcze@163.com (Z.C.); xuyaxin1000@126.com (Y.X.); insravo@foxmail.com (J.R.); w27923780rasj@163.com (J.W.); fly.zf@163.com (F.Z.)

**Keywords:** stress, flexible substrate, microspring structure, RF MEMS switch, substrate bending

## Abstract

A novel stress suppression design for flexible RF MEMS switches has been presented and demonstrated through theoretical and experimental research to isolate the stress caused by substrate bending. An RF MEMS switch with an S-shaped microspring structure was fabricated by the two-step etching process as a developmental step toward miniaturization and high reliability. The RF MEMS switches with an S-shaped microspring exhibited superior microwave performance and stable driving voltage under different substrate curvatures compared to the conventional non-microspring switches, demonstrating that the bending stress is successfully suppressed by the S-shaped microspring and the island structure. Furthermore, this innovative design could be easily extended to other flexible devices.

## 1. Introduction

Recently, liquid crystal polymer (LCP) based devices have been extensively applied to the field of communications [1,2,3,4], radar [5,6,7,8], wearable devices [9,10,11,12], biomedical applications [13,14,15,16] and sensors [17,18,19,20], benefiting from their inherent flexibility and improved electrical material properties. As one of the common RF devices, an RF MEMS switch is indispensable in an RF system. We have presented the multi-physical modeling of bending characteristics to predict the drift of the performance with substrate bending in previous works [21,22,23]. However, it is a fact that the performance of the flexible switch is deteriorated because it is affected by the bending stress when it is conformed to a curved surface. It is critical to design a low packaging stress structure for keeping the substrate as flat as possible under bending conditions.

Many methods have been reported to reduce the packaging stress. In 2015, Schröder S et al. proposed a stress minimized method by double-sided wire bonding [24]. However, double-sided wire bonding is too complicated for mass production. In 2016, Yongcun Hao et al. presented a circular disk with eight L-shaped elastic beams to isolate the stress from out-of-plane deformation based on the silicon substrate, but this stress isolation structure cannot be directly migrated to substrates other than the SOI substrate [25]. In 2019, Bowen Xing et al. reported a single center anchor packaging stress isolation structure for Si-based MEMS devices [26]. Nevertheless, this structure was separated from the rigid substrate, which is detrimental to miniaturization and reliability. Few papers have focused on optimizing the bending stress for flexible devices, aiming at self-packaging, which is closely related to the performance of flexible devices. 

In this paper, for the first time, we proposed an S-shaped microspring and an island structure to minimize the stress induced by substrate bending. The flexible LCP not only acts as a substrate to support the devices but also isolates stress from the substrate bending. The experimental results are consistent with the theoretical analysis and the S-shaped microspring switch successfully maintains superior microwave performance and stable driving voltage under different substrate curvatures compared to the conventional non-microspring switch, demonstrating that the innovative structure exhibits the function of suppressing stress from substrate bending.

## 2. Theoretical Model

**Figure 1:**
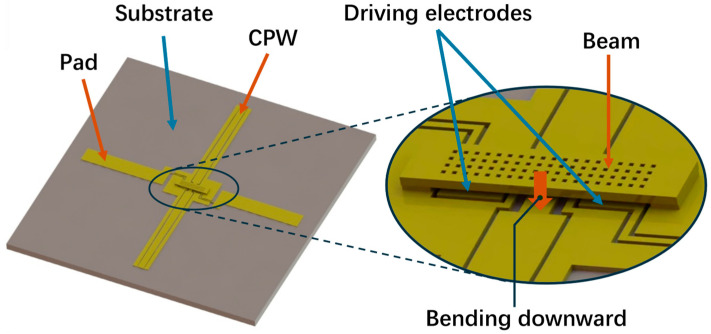
A model of an electrostatic double-clamped switch.

As shown in Figure 1, for the conventional electrostatic double-clamped switch, the driving electrodes are symmetrically located on each side of the double-clamped beam. When the voltage is applied to the driving electrodes in the on state, the electrostatic force is generated between the beam and the driving electrodes. Hence, the beam bends downward under electrostatic force until it reaches the driving voltage. Meanwhile, the beam contacts with the CPW and shifts the switch to the off state. As the flexible substrate bends upward, the gap between the beam and the substrate decreases, generating stress and deteriorating the performance.

**Figure 2:**
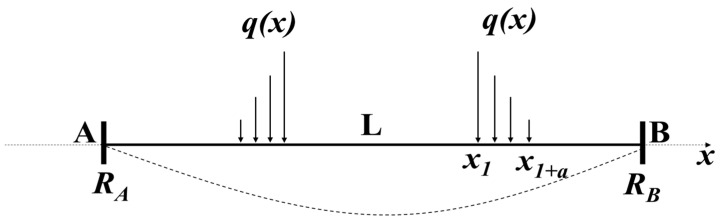
Stretched line model of the double-clamped switch.

When the substrate is flat, the double-clamped beam is simplified into a stretched line to deduce the spring constant and the stretched line model is shown in Figure 2, where *x*_1_ to *x*_1*+a*_ is the location of the right driving electrode and the *q*(*x*) is the load of the force. When the electrostatic force is generated by applying voltage to the driving electrodes, the spring constant *k* could be calculated by the superposition principle.

(1) $$k=\frac{48EI\int_{x_{1}}^{x_{1}+a}q(z)dz}{-\int_{x_{1}}^{x_{1}+a}q(z)(L^{3}-6L^{2}z+9Lz^{2}-4z^{3})dz}$$

(2) $$I=\frac{wt^{3}}{12}$$

where *E* is Young’s modulus; *I* is the rotational inertia; and *L*, *t* and *w* are the length, thickness and width of the double-clamped beam, respectively.

Considering that the driving electrodes are symmetrically located on each side of the beam, the driving voltage can be obtained by:

(3) $$V=\sqrt{\frac{L^{3}g^{3}k}{{\left(L+a\right)}^{3}\varepsilon_{0}\varepsilon_{r}Lw}}$$

where a is the closer distance between the beam and the driving electrodes and *g* is the distance between the beam and the driving electrodes.

When the residual stress of the beam is greater than the critical force, the beam would buckle. The variation of compressive force can be expressed as:

(4) $$\Delta P=\frac{Ewt(1-\varepsilon )\Delta L}{L}$$

where ε is the Poisson ratio and ΔL is the variation of the length of the beam.

When the substrate bends upward, the gap between the beam and driving electrodes decreases resulting from the deformation of the flexible substrate and the beam, which has a direct influence on the driving voltage.

**Figure 3:**
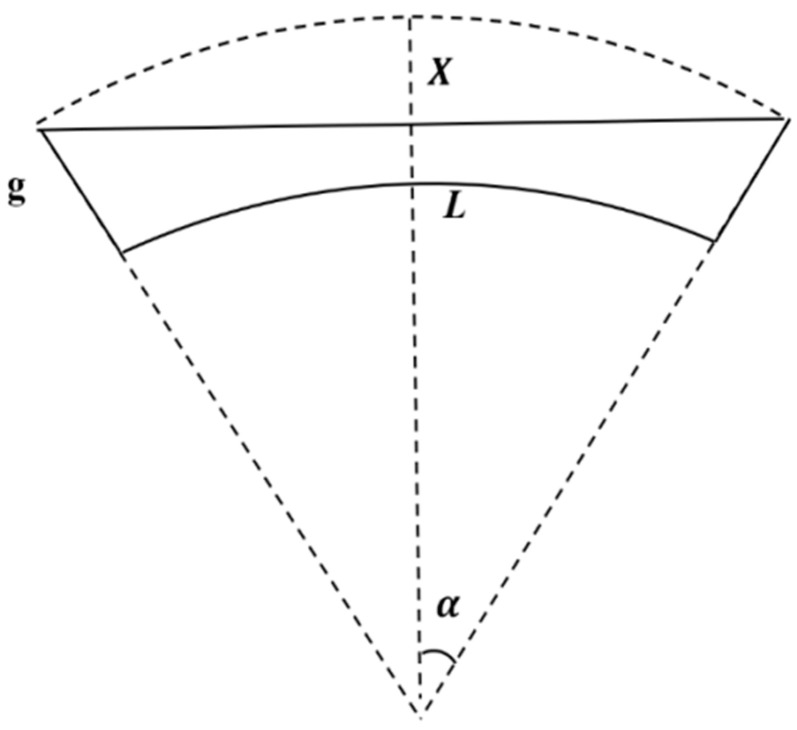
The two-dimension model of the double-clamped beam under the bending condition.

As shown in Figure 3, *L* is the length of the beam, *g* is the initial gap between the beam and the substrate, and *R* is the radius of the flexible bending substrate and could be expressed by:

(5) $$R=\frac{90^{{}^{\circ}}L}{\pi \alpha }$$

The variation in the distance between the beam and the substrate is:

(6) $$X=(R+g)\left[1-\cos\left(\frac{90^{{}^{\circ}}L}{\pi R}\right)\right]$$

If the beam remains flat when the substrate is bending, the variation in the distance between the beam and the substrate could be deduced by:

(7) $$X^{\prime }=g-(R+g)\cos\left(\frac{90^{{}^{\circ}}L}{\pi R}\right)+\sqrt{R^{2}-{\left(a-\frac{L}{2}\right)}^{2}}$$

Therefore, when the substrate is bending upward, the equation of the driving voltage could be further expressed by the following formula considering the influence from $X^{\prime }$:

(8) $$V=\sqrt{\frac{L^{3}{\left(g-X^{\prime }\right)}^{3}k}{{\left(L+a\right)}^{3}\varepsilon_{0}\varepsilon_{r}Lw}}$$

Besides, when the substrate bends, the double-clamped beam deforms under the tensile stress from both the substrate and the beam. Therefore, the tensile force *P* under different bending curvatures could be deduced by:

(9) $$P=\frac{Ewt(1-\varepsilon )\left[2(R+g)\sin\left(\frac{90^{{}^{\circ}}L}{\pi R}\right)-L\right]}{L}$$

**Figure 4:**
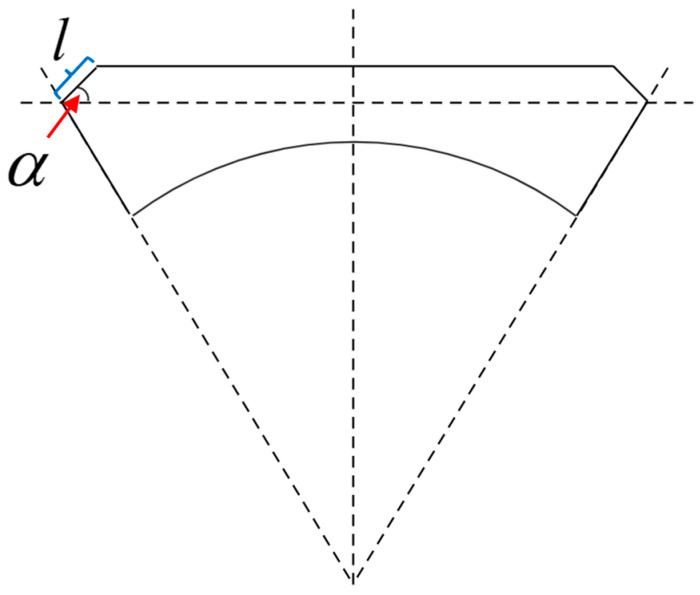
The 2D model of the beam without a spring structure under bending.

As shown in Figure 4, the beam would buckle when the tensile force *P* is less than the residual strain. Therefore, the symmetrical beam structure can be expressed by the deflection equation *u*(*x*) as:

(10) $$\left\{\begin{array}{ll} u(x)=\tan(\alpha )x & \;(0<x<l\cos(\alpha ))\\ u(x)=l\sin(\alpha ) & \;\left(l\cos(\alpha )<x<\frac{L}{2}\right) \end{array}\right.$$

where *l* is the length of the sloping part and *a* is the angle between the sloping part and the flat part. Considering that it is relative to the tensile force *P* under different bending radius, the relationship of *a* and *P* could be expressed by:

(11) $$\alpha (P)=\alpha_{0}-k_{\alpha }\sqrt{\frac{1}{P_{0}-P}}\;(0<P<P_{0})$$

where $a_{0}$ is the maximum angle only caused by the residual stress, *k_a_* is the coefficient, and *P*_0_ is the residual force.

By substituting the deflection equation into the revised driving voltage equation, the driving voltage under different bending radii without a substrate microspring can be obtained by:

(12) $$V=\sqrt{\frac{L^{3}{\left[g+l\sin(\alpha )-X^{\prime }\right]}^{3}k}{{\left(L+a\right)}^{3}\varepsilon_{0}\varepsilon_{r}Lw}}$$

**Figure 5:**
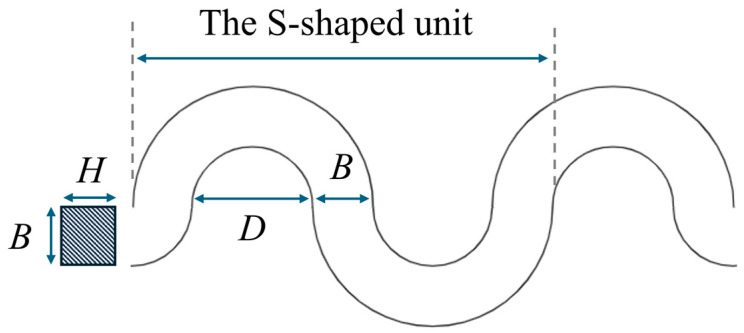
The 2D model of the S-shaped microspring.

As shown in Figure 5, *H* is the thickness, *B* is the line width, and *D* is the gap width. For the S-shaped microspring, the spring constant in tension *k_s_* and vertical spring constant *k_v_* can be respectively expressed as:

(13) $$k_{s}=\frac{2EHB^{3}}{3n\pi {\left(D+B\right)}^{3}}$$

(14) $$k_{v}=\frac{1}{\left(\begin{array}{l} \frac{2\pi n}{GI_{\rho }}{\left(B+\frac{D}{2}\right)}^{3}+\frac{\pi {\left(B+\frac{D}{2}\right)}^{3}}{2EI}[1+3^{2}+5^{2}+\ldots +{\left(4n-1\right)}^{2}]+\\ \frac{\pi {\left(B+\frac{D}{2}\right)}^{3}}{2GI_{\rho }}[1+3^{2}+5^{2}+\ldots +{\left(4n-1\right)}^{2}] \end{array}\right)}$$

(15) $$G=\frac{2E}{\left(1+\varepsilon \right)}$$

where *E* is the Young’s modulus of the material, *n* is the number of the S-shaped unit and $I_{\rho }$ is the polar moment of inertia.

The variation of the S-shape microspring length $\Delta L_{s}$ can be obtained by:

(16) $$\Delta L_{s}=L_{s}-2R\sin\left(\frac{L_{s}}{2R}\right)$$

where $L_{s}$ is the initial length of the S-shape microspring.

Therefore, the stress of the S-shape microspring *T* can be calculated by:

(17) $$T=\frac{k_{s}\Delta L_{s}}{HB}=\frac{2EB^{2}}{3n\pi {\left(D+B\right)}^{3}}\left[L_{s}-2R\sin\left(\frac{90^{{}^{\circ}}L_{s}}{\pi R}\right)\right]$$

## 3. Materials and Tools

The material of the substrate is single-sided copper-clad LCP and the functional layers such as the CPW line, anchor and beam are made up of Au. 

The finite element method by COMSOL 6.2 software is used to analyze the mechanisms of the flexible MEMS switches with the non-microspring and the S-shaped microspring. Ansys Electronics Desktop 2022 R1 is used to simulate the microwave characteristics of the two types of switches.

The different bending curvature frames of 0 m^−1^, 20.0 m^−1^, 22.2 m^−1^, 25.0 m^−1^, and 28.6 m^−1^ are designed to provide the test conditions. The driving voltage and S parameters are measured by an adjustable DC source (UNIT, Dongguan, China), a vector network analyzer (Keysight Technologies, Santa Rosa, CA, USA), and a probe station (Cascade Microtech, Beaverton, OR, USA) with two 150 µm pitch G/S/G probes.

## 4. Design and Simulation

**Figure 6:**
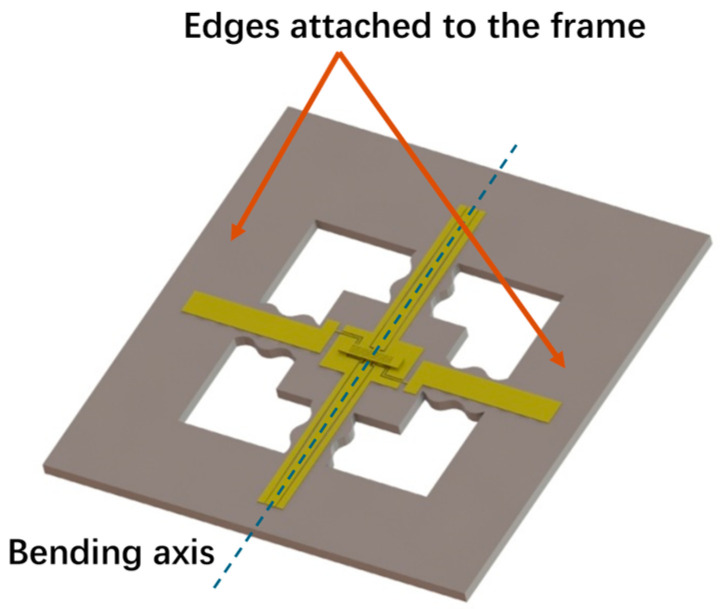
The design of the S-shaped microspring structure.

The RF MEMS switch with an S-shaped microspring structure, which has a low stress package, is designed as in Figure 6. The two edges where the pads are located are fixed and attached to the bending curvature frame, enabling the structure to bend along the CPW. The four cavities create four S-shaped microsprings in the substrate, which turn the central part of the substrate into an island structure. The spring constant of the S-shaped microspring is lower than that of the central island structure. Therefore, the deformation in bending is mainly centralized in the S-shaped microspring when the substrate is curved, which keeps the central island structure almost flat under different curvatures. Thus, the effect of substrate curvature on the switch is reduced, significantly maintaining the outstanding switch performance.

The low stress package size is designed to be 6 mm × 6 mm and the geometric parameters of the double-clamped beam switch are listed in Table 1. 

**Figure 7:**
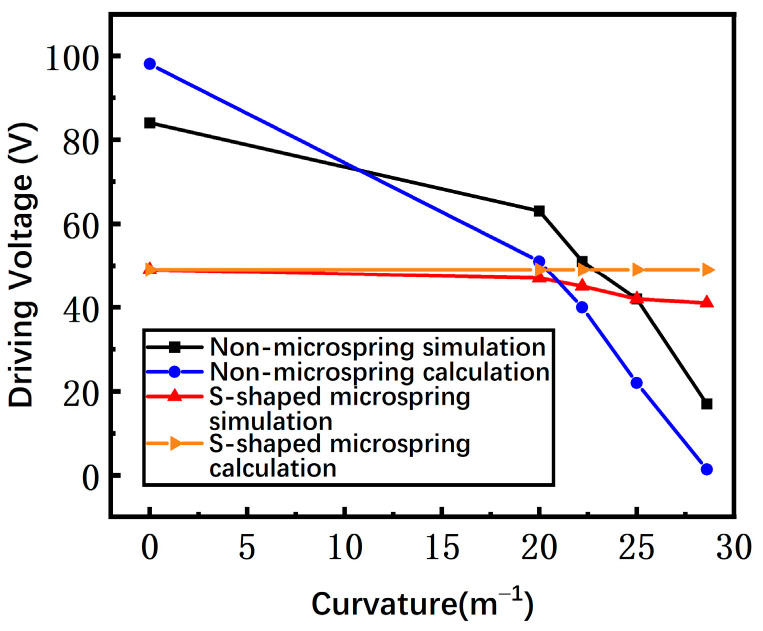
The simulated and calculated driving voltages of switches with non-microsprings and S-shaped microsprings as the substrate bending curvature increases.

As the curvature rises, the stress of the switch is increased and the gap between the beam and the driving electrode is reduced. The driving voltage is related to the stress and the gap, and the overall effect on the driving voltage is decreasing. The simulated driving voltages of the switches with a non-microspring and an S-shaped microspring versus the curvature are shown in Figure 7. For a non-microspring switch, the driving voltage declines dramatically. However, the variation of the S-shaped microspring is relatively flat, demonstrating that the stress and the gap of the switch are nearly unchanged. Therefore, the S-shaped microspring and the island structure indeed efficiently reduce the stress caused by the substrate bending, which is consistent with the theoretical predictions. 

**Figure 8:**
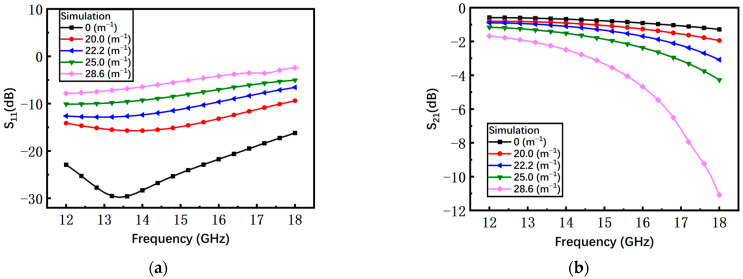
The simulated results of the non-microspring switch in the on state. (**a**) Return loss S_11_; (**b**) insertion loss S_21_.

**Figure 9:**
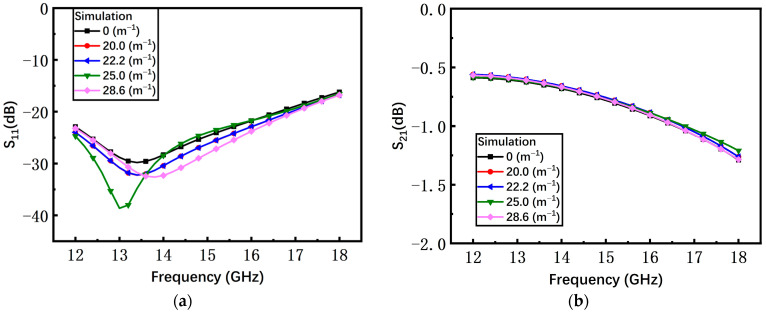
The simulated results of the S-shaped microspring switch in the on state. (**a**) Return loss S_11_; (**b**) insertion loss S_21_.

As shown in Figure 8, with the increase of the curvature, the simulated return loss S_11_ and insertion loss S_21_ of the switch with the non-microspring are continuously degraded. However, since the gap of the switch with the S-shaped microspring barely changes, the S-parameters of different curvatures almost coincide with the curvature of 0 m^−1^ as shown in Figure 9.

According to Equation (6), as the curvature increases, the gap of the non-microspring switch between the beam and the substrate reduces, leading to the deterioration in microwave performance. The S-shaped microspring keeps the central island structure flat under different curvatures and reduces the stress caused by the substrate bending, maintaining the high microwave performance. Hence, the simulation results are consistent with the tendency from the theoretical analysis.

**Figure 10:**
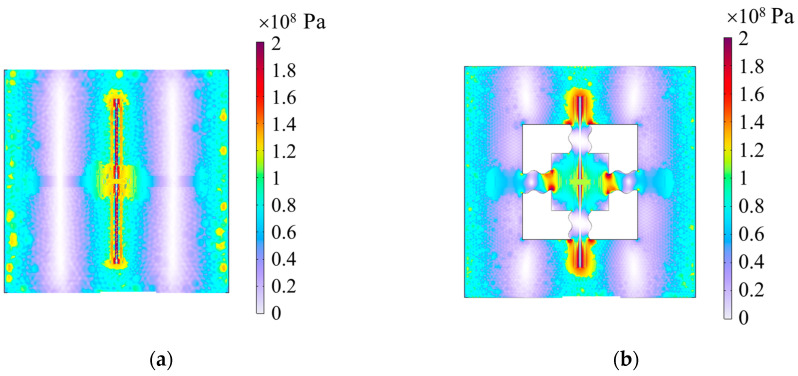
The simulated results of the stress distribution. (**a**) Non-microspring structure; (**b**) S-shaped microspring structure.

The simulation results of the stress distribution are shown in Figure 10. The stress of the non-microspring structure is concentrated in the switch. However, for the S-shaped microspring structure, the stress is centralized in the S-shaped microspring, effectively reducing the bending influence on the switch.

## 5. Fabrication

**Figure 11:**
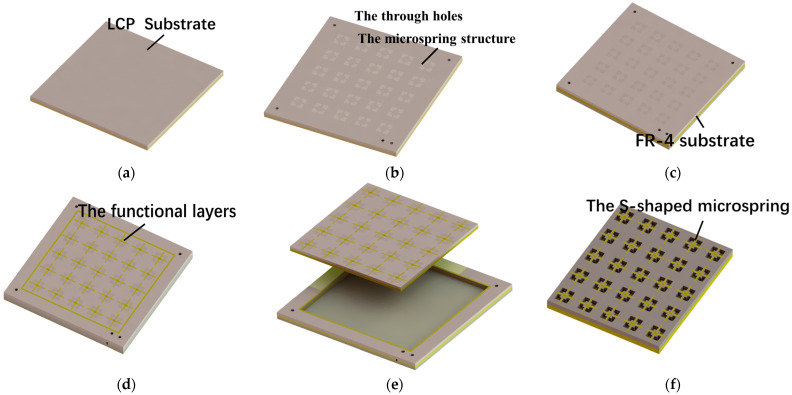
The fabrication of S-shaped microspring structures. (**a**) sample preparation; (**b**) laser etching; (**c**) temporary bonding; (**d**) preparation of functional layers; (**e**) debonding; (**f**) the backside etching to get the S-shaped microspring.

**Figure 12:**
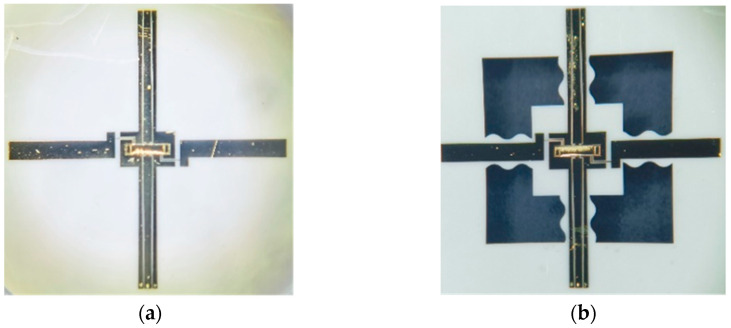
An optical image of two types of switches. (**a**) A switch with a non-microspring structure; (**b**) a switch with an S-shaped microspring structure.

The fabrication process of the switch with an S-shaped microspring structure is shown in Figure 11. The microspring structures are firstly etched by laser and then bonded to a rigid substrate by temporary bonding to guarantee the substrate’s flatness [27]. After that, the CPW line is formed by traditional lithography, sputtering, plating, and etching. The photoresist is patterned on the CPW line to act as a sacrificial layer and could easily be removed in the following steps. Afterward, the 2 μm thick beam is fabricated on the organic sacrificial layer by optimizing the plating time, plating current density, and plating electrolyte to achieve a good elasticity modulus. Next, the substrate microspring structures could be formed by backside wetting etching. At last, the sample is debonded and the sacrificial layer is removed by wetting treatment combined with supercritical drying. Meanwhile, the profile of the beam is kept and the fabrication of the switch is finished. Optical images of the two types of switches are shown in the Figure 12. 

## 6. Results and Discussion

**Figure 13:**
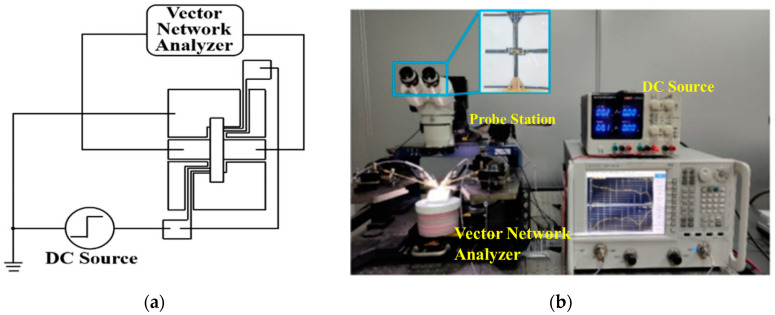
(**a**) The test circuit for the driving voltage and the microwave performance. (**b**) The measurement setup for testing the as-fabricated switches.

The measurement setup for testing the fabricated switches is shown in Figure 13. The switch is conformed to the test frame to measure the driving and microwave performance under different substrate bending conditions. 

**Figure 14:**
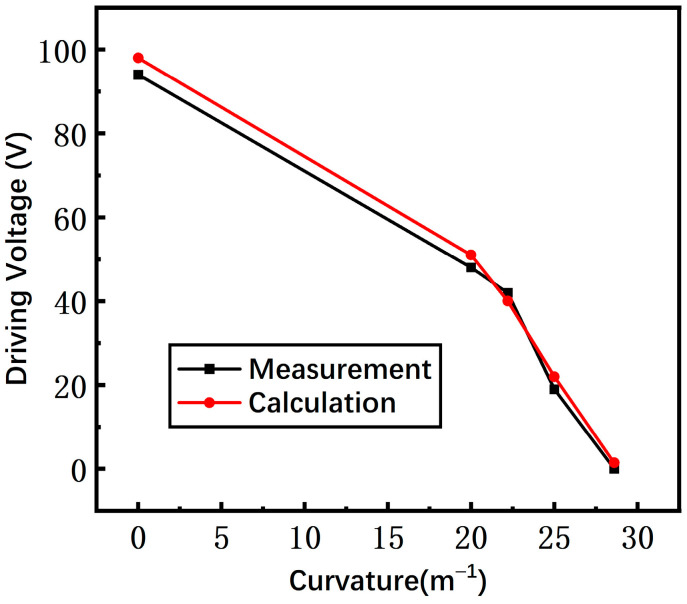
The measured and calculated driving voltage of a switch with a non-microspring structure.

The measured and calculated driving voltages of the switch with the non-microspring structure are shown in Figure 14. The two curves show the same variation trend that the driving voltage decreases as the substrate curvature increases since the gap between the beam and driving electrodes are dramatically reduced. Meanwhile, the measured results are in satisfactory agreement with the calculated results, within an error of 16%.

**Figure 15:**
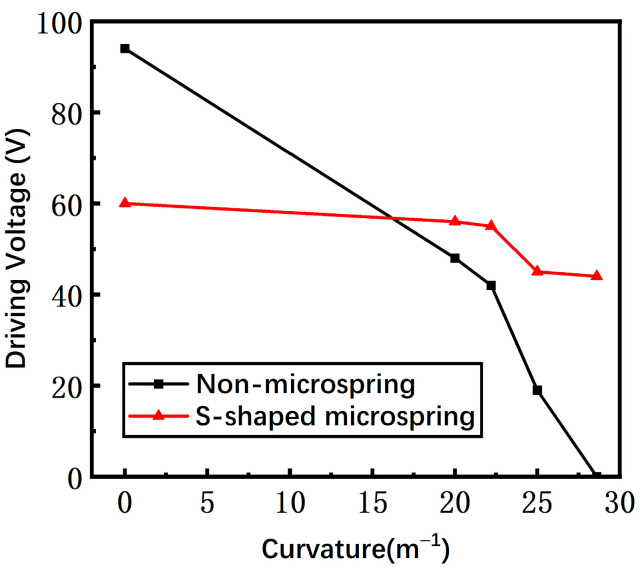
The measured driving voltage of non-microspring and S-shaped microspring switches as the bending curvature increases.

The measured driving voltages of switches with a non-microspring and an S-shaped microspring are shown in Figure 15. The non-microspring switch suffers the whole bending effect, and thus the driving voltage drops from 94 V to 0 V when the substrate curvature rises from 0 m^−1^ to 28.6 m^−1^. However, the S-shaped microspring switch has less variation compared to the conventional non-microspring switch, decreasing from 60 V to 40 V with a substrate curvature range of 0 m^−1^ to 28.6 m^−1^. Therefore, the gap of the S-shaped microspring switch is almost unaffected by the substrate curvature, confirming that the S-shaped microspring and the island structure are efficient in suppressing the bending stress. 

**Figure 16:**
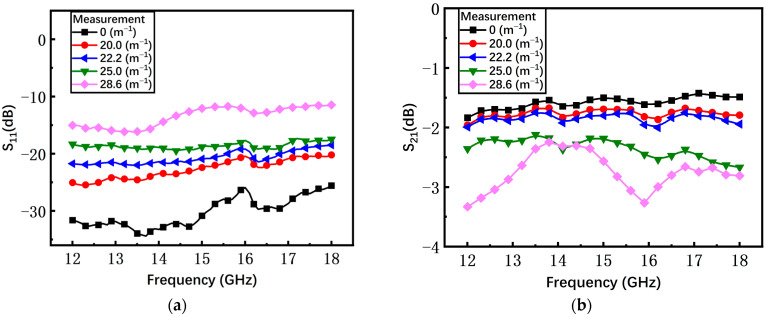
The measured results of the non-microspring switch in the on state. (**a**) Return loss S_11_; (**b**) insertion loss S_21_.

As shown in Figure 16a, with the increasing curvature, the average measured return loss S_11_ of the non-microspring switch has an obvious degradation from −30 dB to −13 dB. When the bending curvature is 28.6 m^−1^, the S_11_ increases by at least 13 dB compared to the initial state. The measured insertion loss S_21_ under different curvatures is shown in Figure 16b and the average S_21_ is degraded by 1.17 dB, from −1.58 dB to −2.75 dB.

**Figure 17:**
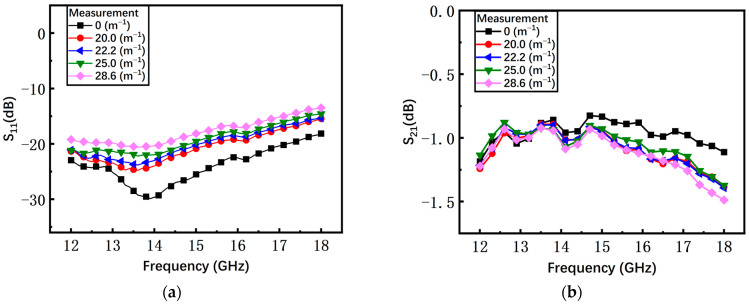
The measured results of the S-shaped microspring switch in the on state. (**a**) Return loss S_11_; (**b**) insertion loss S_21_.

The S parameters of the S-shaped microspring switch are shown in Figure 17. With the increase of bending curvature from 0 m^−1^ to 28.6 m^−1^, the average return loss S_11_ deteriorates from −24 dB to −18 dB and the insertion loss S_21_ is almost invariable. As a result, the fluctuation of the S-shaped microspring switch is lower than the non-microspring switch, validating that the bending stress is successfully suppressed by the S-shaped microspring and the island structure.

**Figure 18:**
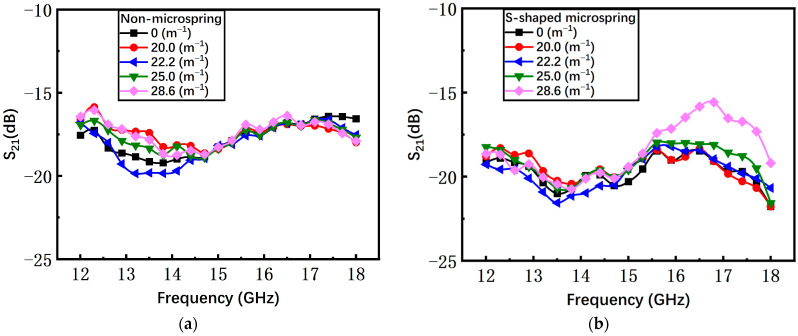
The isolation S_21_ of two types of switches in the off state. (**a**) the non-microspring switch; (**b**) the S-shaped microspring switch.

The isolation S_21_ of the two switches with different bending curvatures in the off state are overlapping as shown in Figure 18. The results are independent of the substrate curvature, since the off state originates from the contact of the beam with the CPW.

The measured results are consistent with the simulated results. For the non-microspring switch, the average error of return loss between the measured and simulated results is within 8.9 dB and the error of insertion loss is 0.68 dB. Correspondingly, the average error of return loss and insertion loss for the S-shaped microspring switch is 4.5 dB and 0.24 dB, respectively. The characteristic impedance of the switch is not a precise 50 Ω, which is a factor causing an error of return loss and insertion loss. Furthermore, the initial height of the non-microspring switch beam is not 2 μm as expected due to the residual stress resulting from the fabrication process, which also affects the S-parameters. In general, the S-shaped microspring and the island structure effectively suppress the bending stress, ensuring the maintenance of an outstanding microwave performance and invariant driving voltage.

## 7. Conclusions

In conclusion, a low self-packaging stress method of producing a flexible RF MEMS switch is presented and verified by the theoretical and experimental results. Apart from being a substrate, the LCP also serves as a stress isolation through the S-shaped microspring and the island structure formed by the cavities. The calculated, simulated, and measured results are consistent with others, and the provided results demonstrate that the S-shaped microspring and the island structure effectively suppress the bending stress, resulting in superior microwave performance and stable driving voltage of the S-shaped microspring switch under different substrate curvatures compared to a conventional non-microspring switch. Hence, the stress suppression design proposed in this paper is a promising choice for the bending environment and is easy to extend to other flexible devices, showing enormous potential for device miniaturization and reliability.

## Figures and Tables

**Table 1:** Geometric parameters of the double-clamped beam switch.

Item	Size (μm)
Width of the beam	190
Length of the beam	960
Thickness of the beam	2
Width of the driving electrode	200
Length of the driving electrode	200
Thickness of the driving electrode	2
The gap between the original beam and driving electrode	2
Width of the cavity	1970
Length of the cavity	1970
Width of the S-shape microspring	664
Length of the S-shape microspring	1015
Package size of the switch	6000 × 6000
Width of the pad	400
Length of the pad	2225

## Data Availability

The original contributions presented in the study are included in the article, further inquiries can be directed to the corresponding author.

## References

[1] You D.W., Fu X., Herdian H., Wang X.L., Narukiyo Y., Fadila A.A., Lee H.J., Ide M., Kato S., Li Z. (2023). A Ka-Band 64-Element Deployable Active Phased-Array TX on a Flexible Hetero Segmented Liquid Crystal Polymer for Small Satellites. IEEE Microw. Wirel. Technol. Lett..

[2] Devi Y.U., Rukmini M.S.S., Madhav B.T.P. (2018). Liquid crystal polymer based flexible and conformal 5G antenna for vehicular communication. Mater. Res. Express..

[3] Anilkumar T., Madhav B.T.P., Rao M.V., Nadh B.P. (2020). Bandwidth reconfigurable antenna on a liquid crystal polymer substrate for automotive communication applications. AEU-Int. J. Electron. C.

[4] Zhou Z.P., Li W.Q., Qian J., Liu W.H., Wang Y.M., Zhang X.J., Yashchyshyn Y., Wang Q.P., Shi Y.P., Zhang Y.F. (2022). Flexible Liquid Crystal Polymer Technologies from Microwave to Terahertz Frequencies. Molecules.

[5] Kim S., Rida A., Lakafosis V., Nikolaou S., Tentzeris M.M. (2019). 77-GHz mmWave antenna array on liquid crystal polymer for automotive radar and RF front-end module. ETRI J..

[6] Zhang X., Liu J., Cai P., Kärnfelt C., Wang X., Ma S.W., Morris J., Zirath H. (2008). Millimeter-wave ultra-wideband bandpass filter based on liquid crystal polymer substrates for automotive radar systems. Microw. Opt. Technol. Lett..

[7] Lee D., Nguyen C. A millimeter-wave dual-band dual-polarization antenna on liquid crystal polymer. Proceedings of the 2014 IEEE Antennas and Propagation Society International Symposium (APSURSI).

[8] Kathuria N., Seet B.C. (2021). 24 GHz Flexible Antenna for Doppler Radar-Based Human Vital Signs Monitoring. Sensors.

[9] Peng X.X., Du C.Z. (2024). A flexible CPW-fed tri-band four-port MIMO antenna for 5G/WIFI 6E wearable applications. AEU-Int. J. Electron. C.

[10] Nadh B.P., Madhav B.T.P., Kumar M.S., Kumar T.A., Rao M.V., Reddy S.S.M. (2022). MEMS-based reconfigurable and flexible antenna for body-centric wearable applications. J. Electromagnet. Wave..

[11] Kim C., Kim J.K., Kim K.T., Yoon Y.K. Micromachined wearable/foldable super wideband (SWA) monopole antenna based on a flexible liquid crystal polymer (LCP) substrate toward imaging/sensing/health monitoring systems. Proceedings of the 2013 IEEE 63rd IEEE Electronic Components and Technology Conference.

[12] Ali Khan M.U., Raad R., Tubbal F., Theoharis P.I., Liu S., Foroughi J. (2021). Bending analysis of polymer-based flexible antennas for wearable, general IoT applications: A review. Polymers.

[13] Lee S.Y., Park K.I., Huh C., Koo M., Yoo H.G., Kim S., Ah C.S., Sung G.Y., Lee K.J. (2012). Water-resistant flexible GaN LED on a liquid crystal polymer substrate for implantable biomedical applications. Nano Energy.

[14] Park R., Lee D.H., Koh C.S., Kwon Y.W., Chae S.Y., Kim C.S., Jung H.H., Jeong J., Hong S.W. (2024). Laser-Assisted Structuring of Graphene Films with Biocompatible Liquid Crystal Polymer for Skin/Brain-Interfaced Electrodes. Adv. Healthc. Mater..

[15] Lee S.E., Jun S.B., Lee H.J., Kim J., Lee S.W., Im C., Shin H.C., Chang J.W., Kim S.J. (2012). A flexible depth probe using liquid crystal polymer. IEEE Trans. Biomed. Eng..

[16] Yun S., Koh C.S., Jecong J., Seo J., Kim S.J. (2019). Remote-controlled fully implantable neural stimulator for freely moving small animal. Electronics.

[17] Kottapalli A.G.P., Tan C.W., Olfatnia M., Miao J.M., Barbastathis G., Triantafyllou M. (2011). A liquid crystal polymer membrane MEMS sensor for flow rate and flow direction sensing applications. J. Micromech. Microeng..

[18] Kottapalli A.G.P., Asadnia M., Miao J.M., Barbastathis G., Triantafyllou M.S. (2012). A flexible liquid crystal polymer MEMS pressure sensor array for fish-like underwater sensing. Smart. Mater. Struct..

[19] Nie M., Yang H.S., Xia Y.H. (2018). Graphene based strain sensor with LCP substrate. IOP Conf..

[20] Redhwan T.Z., Alam A.U., Haddara Y.M., Howlader M.M.R. (2018). Copper and liquid crystal polymer bonding towards lead sensing. Jpn. J. Appl. Phys..

[21] Han L., Chen L.J., Qin R.J., Wang K., Zhang Z.Q., Nie M., Huang X.D. (2020). Multi-Physical Models of Bending Characteristics on the Double-Clamped Beam Switch for Flexible Electronic Devices Application. Sensors.

[22] Han L., Yu Y., Qin R.J., Zhang Z.Q., Su S. (2019). Static Modeling of Bending Characteristics on V-Shaped Beam Actuator Based on Flexible Substrate. IEEE Trans. Electron. Dev..

[23] Han L., Gao X.F. (2019). Modeling of Bending Characteristics on Micromachined RF MEMS Switch Based on LCP Substrate. IEEE Trans. Electron. Dev..

[24] Schröder S., Niklaus F., Nafari A., Westby E.R., Fischer A.C., Stemme G., Haasl S. (2015). Stress-minimized packaging of inertial sensors by double-sided bond wire attachment. J. Microelectromech. Syst..

[25] Hao Y.C., Yuan W.Z., Xie J.B., Shen Q., Chang H.L. (2016). Design and verification of a structure for isolating packaging stress in SOI MEMS devices. IEEE Sens. J..

[26] Xing B.W., Zhou B., Wang J., Hou B., Li X., Wei Q., Zhang R. A novel packaging stress isolation chip for MEMS devices. Proceedings of the 2019 IEEE SENSORS.

[27] Wang K., Han L., Xu Y.X., Chai Z.E., Ren J.W., Wang J., Wang Z.Y., Qin M., Zhao F. The Control of Substrate Flatness for Flexible LCP-based Devices by Temporary Bonding and Two-step Etching Method. Proceedings of the 2023 Second International Conference on Electrical, Electronics, Information and Communication Technologies (ICEEICT).

